# Imaging the Facial Nerve: A Contemporary Review

**DOI:** 10.1155/2013/248039

**Published:** 2013-05-23

**Authors:** Sachin Gupta, Francine Mends, Mari Hagiwara, Girish Fatterpekar, Pamela C. Roehm

**Affiliations:** ^1^Department of Otolaryngology, New York University School of Medicine, New York, NY 10016, USA; ^2^Department of Radiology, New York University School of Medicine, New York, NY 10016, USA

## Abstract

Imaging plays a critical role in the evaluation of a number of facial nerve disorders. The facial nerve has a complex anatomical course; thus, a thorough understanding of the course of the facial nerve is essential to localize the sites of pathology. Facial nerve dysfunction can occur from a variety of causes, which can often be identified on imaging. Computed tomography and magnetic resonance imaging are helpful for identifying bony facial canal and soft tissue abnormalities, respectively. Ultrasound of the facial nerve has been used to predict functional outcomes in patients with Bell's palsy. More recently, diffusion tensor tractography has appeared as a new modality which allows three-dimensional display of facial nerve fibers.

## 1. Introduction

Imaging plays an important role in the evaluation of facial nerve disorders. The facial nerve has a complex anatomical course, and dysfunction can be due to congenital, inflammatory, infectious, traumatic, and neoplastic etiologies. Computed tomography is useful for identifying bony abnormalities of the intratemporal facial nerve, which can occur with congenital malformations, trauma, and cholesteatoma. Magnetic resonance imaging (MRI) is useful for identifying soft tissue abnormalities around the facial nerve, as seen in inflammatory disorders, neoplasms, and hemifacial spasm. Facial nerve ultrasound has been used in a recent study to predict functional outcomes in Bell's palsy [1]. Diffusion tensor (DT) tractography, which uses MRI to make three-dimensional (3D) reconstructions of the facial nerve, has recently been developed. This technique has been shown to be potentially useful in the identification displacement of cranial nerve fibers by vestibular schwannomas [2]. In all cases, choice of the imaging modality utilized should be determined by specifics of the patient's symptoms and the differential diagnosis. In this paper we describe the development and anatomy of the facial nerve, then radiographic techniques used in facial nerve evaluation, and finally the pathologic entities that affect the facial nerve. 

## 2. Development and Anatomy of the Facial Nerve

The facial nerve is composed of motor, sensory, and parasympathetic fibers. Complete separation of the facial and acoustic nerves and development of the nervus intermedius (or nerve of Wrisberg) occurs by 6 weeks of gestation. By the 16th week, the neural connections are completely developed. The bony facial canal develops until birth, enclosing the facial nerve in bone throughout its course except at the facial hiatus (the site of the geniculate ganglion) in the floor of the middle cranial fossa [3, 4]. The only difference between the anatomy of the facial nerve in infants compared with adults is in the region of the stylomastoid foramen. As the mastoid tip develops, the extratemporal facial nerve is positioned in a more inferior and medial position.

**Figure 1:**
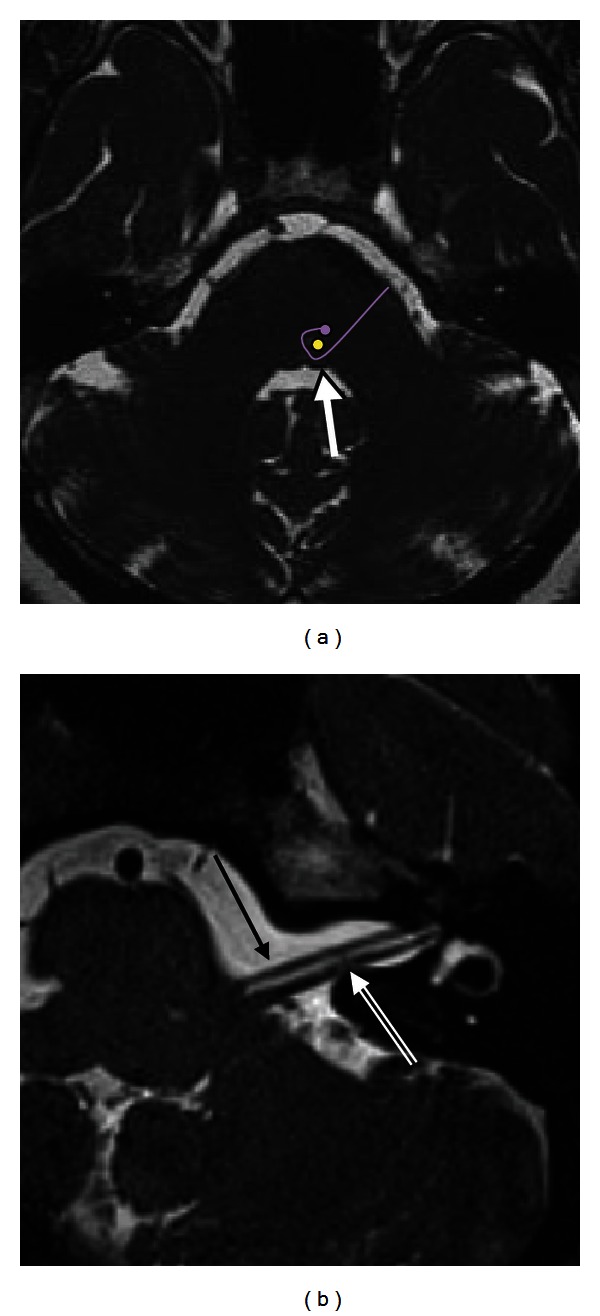
Normal facial nerve on MRI. (a) Axial CISS image at the level of the pons demonstrates the facial colliculus (arrow) seen as a small bump along the anterior wall of the fourth ventricle. This is formed by the motor tracts of the facial nerve (purple curved line) coursing around the abducens nucleus (yellow dot). (b) Axial CISS sequence of the left CPA and IAC demonstrates the normal cisternal and intracanalicular segments of the left CN VII (solid arrow), anterior to CN VIII (double lined arrow).

Facial motor fibers originate from cell bodies located in the precentral and postcentral gyri of the frontal motor cortex. These fibers travel in the posterior limb of the internal capsule inferiorly to the caudal pons. There, the motor fibers supplying the facial musculature beneath the brows cross the midline to reach the contralateral motor nucleus in the reticular formation of the lower pons (anterior to the fourth ventricle). The majority of motor fibers that supply the musculature of the forehead also cross the midline; however, a few fibers do not, instead traveling in the ipsilateral motor nucleus. Thus, muscles of the forehead receive innervation from both sides of the motor cortex, and so forehead-sparing facial paralyses can be indicative of a central etiology. The motor fibers the pass dorsally, loop medial-to-lateral around the abducens nucleus, and create the facial colliculus, which bulges into the floor of the fourth ventricle (Figure 1). This loop of the facial nerve forms the internal genu of the facial nerve [5, 6].

The nervus intermedius contains sensory, special sensory and parasympathetic fibers. It provides sensation to the posterior concha and external auditory canal. The nervus intermedius' special sensory fibers supply taste sensation to the anterior two-thirds of the tongue. The afferent fibers synapse with cell bodies in the geniculate ganglion at the first genu of the facial nerve. These sensory afferents then join the parasympathetic fibers, passing via the nervus intermedius to the nucleus tractus solitarius in the medulla. The parasympathetic portion of the nervus intermedius originates in the superior salivatory nucleus in the dorsal pons and provides the secretomotor function of the ipsilateral lacrimal gland, submandibular glands, sublingual glands, and minor salivary glands. 

 Both the motor root of the facial nerve and the nervus intermedius leave the brainstem near the dorsal pons at the pontomedullary junction (the cisternal segment of the facial nerve). Within the cerebellopontine angle (CPA), the nerve travels anterolaterally into the porus acousticus of the internal auditory canal (IAC), anterior to the vestibulocochlear nerve (Figure 1(b)). This segment is 24 mm [7]. The nervus intermedius either joins the motor root as it emerges from the brainstem or near the meatus of the IAC [8]. The facial nerve runs in the anterior-superior quadrant of the IAC. At the lateral end of the IAC, a horizontal segment of bone (the transverse of falciform crest) separates the facial nerve from the cochlear nerve inferiorly. Within this area of the IAC, a vertical segment of bone (Bill's bar) separates the facial nerve from the posteriorly located superior vestibular nerve. 

The anterior inferior cerebellar artery (AICA) arises from the basilar artery near the junction of the pons and medulla. The AICA can have a variable course and territory. The AICA runs within the IAC and is frequently in proximity with the nerve within the IAC. In some cases, the AICA may run in the IAC between the facial and vestibulocochlear nerve [9]. The blood supply to this region of the facial nerve is the labyrinthine artery, a branch of AICA. 

**Figure 2:**
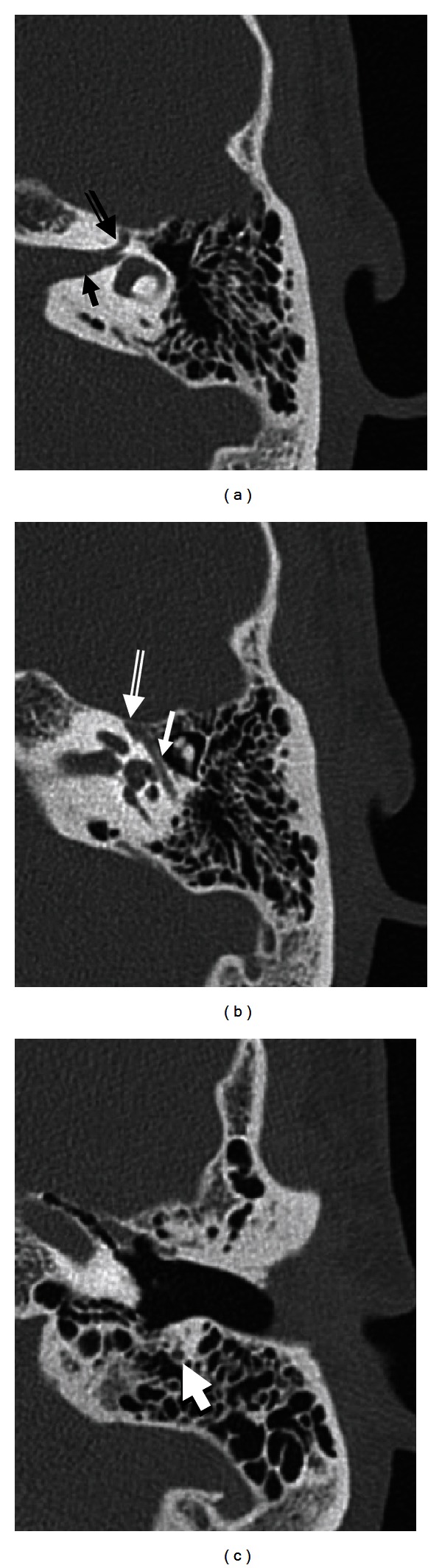
Normal facial nerve canal on CT. Axial temporal bone CT images demonstrate the intracanalicular (solid arrow in (a)), labyrinthine (double lined arrow in (a)), geniculate ganglion (double lined arrow in (b)), tympanic (solid arrow in (b)), and mastoid (arrow in (c)) segments of the facial nerve.

**Figure 3:**
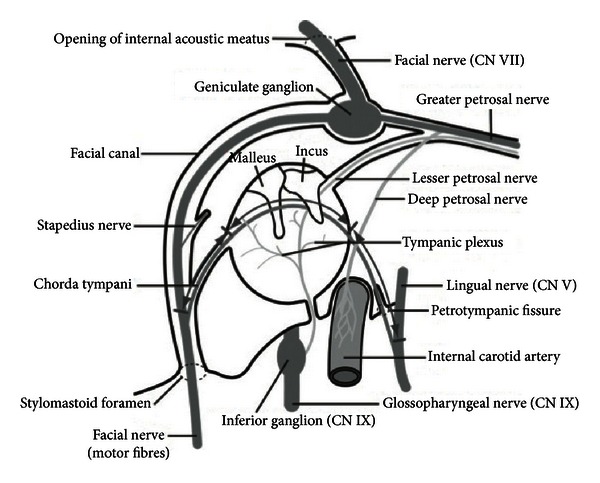
Diagram of the course of the intratemporal facial nerve from the fundus of the IAC to the stylomastoid foramen [10].

 The bony facial nerve canal (or fallopian canal) begins as the facial nerve exits the IAC at the fundus. The major blood supply for the facial nerve proximally within the canal is the superficial petrosal artery, a branch of the middle meningeal artery. The stylomastoid artery supplies the fallopian canal distally [11]. The bony canal has three segments: labyrinthine, tympanic, and mastoid (Figures 2 and 3). The labyrinthine segment runs from the fundus of the IAC to the geniculate ganglion. It is both the narrowest (<0.7 mm diameter) and shortest (3–5 mm length) segment of the facial nerve. The labyrinthine segment travels anterior-laterally from the IAC and superior to the cochlea until it reaches the geniculate ganglion.

While the geniculate ganglion is typically covered by bone, in up to 18% of cases the ganglion is in direct contact with the dura of the middle cranial fossa [8]. The first branch of the facial nerve, the greater superficial petrosal nerve (GSPN), exits anteriorly from the geniculate ganglion. The GSPN carries preganglionic parasympathetic fibers from the superior salivatory nucleus, and runs along the anterior surface of the temporal bone into the pterygoid canal (Vidian canal). It synapses at the pterygopalatine ganglion in the pterygopalatine fossa. Postganglionic parasympathetic fibers then join the maxillary nerve to innervate the lacrimal gland and small salivary glands in the nose and palate. At the geniculate ganglion, the facial nerve makes a 75 degree turn posteriorly to become the tympanic segment (the first or anterior genu).

 The tympanic segment runs from the geniculate ganglion to the second (or posterior) genu [12]. Within the tympanic cavity, the facial nerve passes medial to the incus. It runs posterior-superior to the cochleariform process, superior and lateral to the oval window, and then inferior to the lateral semicircular canal. Bony dehiscence of the facial nerve canal is seen in 41–75% of people within this segment [4, 11, 12]. At the pyramidal process, the tympanic segment turns inferiorly at a 95°–125° angle (at the second genu) to become the mastoid or vertical segment [12].

 The mastoid segment of the facial nerve runs posteromedially along the external auditory canal to its exit from the temporal bone at the stylomastoid foramen (13 mm in adults) [12]. Two branches arise from the mastoid segment: the nerve to the stapedius and the chorda tympani (Figure 3). The stylomastoid foramen arises between the styloid process anteriorly and the mastoid process posteriorly. The nerve exits the temporal bone at the stylomastoid foramen, entering the substance of the parotid gland. 

Extratemporally, the facial nerve separates into two main branches at the pes anserinus: the temporofacial branch and the cervicofacial branch. Within the parotid gland, these branches further divide into five main branches that supply the facial musculature: temporal (or frontal), zygomatic, buccal, marginal mandibular, and cervical. 

## 3. Imaging Techniques for the Evaluation of the Facial Nerve

Imaging of the facial nerve should be tailored to both the suspected pathology and clinical localization of the lesion along the nerve's course. Typically, if a facial palsy is localized to the cisternal or intracanalicular segments of the facial nerve or the pontine nuclei, contrast-enhanced MRI is indicated. If the lesion can be localized to the mastoid, tympanic, or labyrinthine segments of the facial nerve, high-resolution temporal bone CT is recommended to evaluate the fallopian canal. Contrast-enhanced MRI should be performed first in cases when the palsy cannot be definitively localized. Both MRI and temporal bone CT are typically performed for the evaluation of tumors involving the facial nerve.

### 3.1. CT

 CT is preferable for imaging the lateral course of the facial nerve from the porus acusticus to the stylomastoid foramen. CT evaluation of the facial nerve lateral to the porus acusticus should include high-resolution temporal bone CT. Temporal bone CT is particularly useful in the evaluation of the caliber and the course of the IAC and bony facial nerve canal in the temporal bone. Erosion and destruction of the facial nerve canal are best depicted with high-resolution temporal bone CT. In addition, CT has the advantage of demonstrating the relationship of the facial nerve canal to normal anatomic landmarks such as the ossicles which are not seen on MR. These relationships are critical during surgical planning. At our institution, we acquire noncontrast 0.3 mm axial slices through the bilateral temporal bones for our raw data, with 0.6 mm thick axial, coronal, and Pöschl reformats through the individual temporal bones. Dose settings are typically 120 kVp, CTDI volume 55.5—62.6, and 140–220 mAs. Administration of intravenous contrast is not part of the standard protocol but may be included if there is a clinical suspicion of a neoplasm or vascular abnormality.

Temporal bone CT can detect deviations in the course and caliber of the intratemporal facial nerve, which can provide key information regarding facial nerve pathology and prove critical in surgical planning for otologic surgery. Bony dehiscences of the fallopian canal can be identified preoperatively, leading to a decreased rate of iatrogenic facial nerve trauma. In cases of aural atresia, the facial nerve can vary in its course, making it more susceptible to injury during atresiaplasty and limiting the diameter of the reconstructed external auditory canal [13]. High-resolution temporal bone CT can also identify temporal bone fractures that violate the facial nerve canal. Using 16- or 64-slice CT, helical mode acquisition, 0.6 mm slice thickness, and 0.3 mm reconstruction increments, Mu et al. [14] found a significant correlation between findings of increased transverse geniculate ganglion diameter on CT and intraoperative identification of fractures involving the geniculate ganglion (*P* < 0.001). All patients with an enlarged geniculate ganglion fossa (transverse diameter 1.9 ± 0.3 mm) were found to have geniculate ganglion fossa fractures during surgical exploration. Two of these patients did not have visible temporal bone fractures on high-resolution temporal bone CT.

### 3.2. MRI

When using CT to evaluate the facial nerve, pathology often can only be inferred by visualization of erosion or destruction of the adjacent bony facial nerve canal. In contrast, MRI visualizes soft tissues well and so is better suited for evaluating soft tissue facial nerve abnormalities. MRI can be used to image the facial nerve from the brainstem to the fundus of the internal auditory canal and to determine the presence of perineural spread from parotid malignancies (Figure 9). At our institution, standard MRI imaging for evaluation of the facial nerve and inner ear pathology includes an IAC protocol with the following sequences: a noncontrast axial 5 mm whole brain T2-weighted sequence; an axial 3 mm, T1-weighted sequence of the IAC angled perpendicular to dorsal aspect of the brainstem; an axial constructive interference in the steady-state (CISS) sequence (heavily T2-weighted sequence, 0.6 mm) from the occipital bone to superior petrous ridge; axial 5 mm whole brain postcontrast FLAIR and T1-weighted sequences; a coronal 4 mm postcontrast T1-weighted sequence of the IAC; and an axial 3 mm T1-weighted fat-saturated sequence of the IAC. 

In high-resolution T2-weighted images or CISS images, the normal facial nerve appears as a hypointense linear structure extending from the brainstem to the IAC, anterior to the vestibulocochlear nerve, surrounded by T2 hyperintense cerebrospinal fluid (Figure 1). The labyrinthine, tympanic, and mastoid segments of the facial nerve are not typically well visualized in noncontrast images. The proximal extracranial portion of the facial nerve in the parotid gland is best visualized with axial high-resolution T1-weighted images using a microscopic coil.

**Figure 4:**
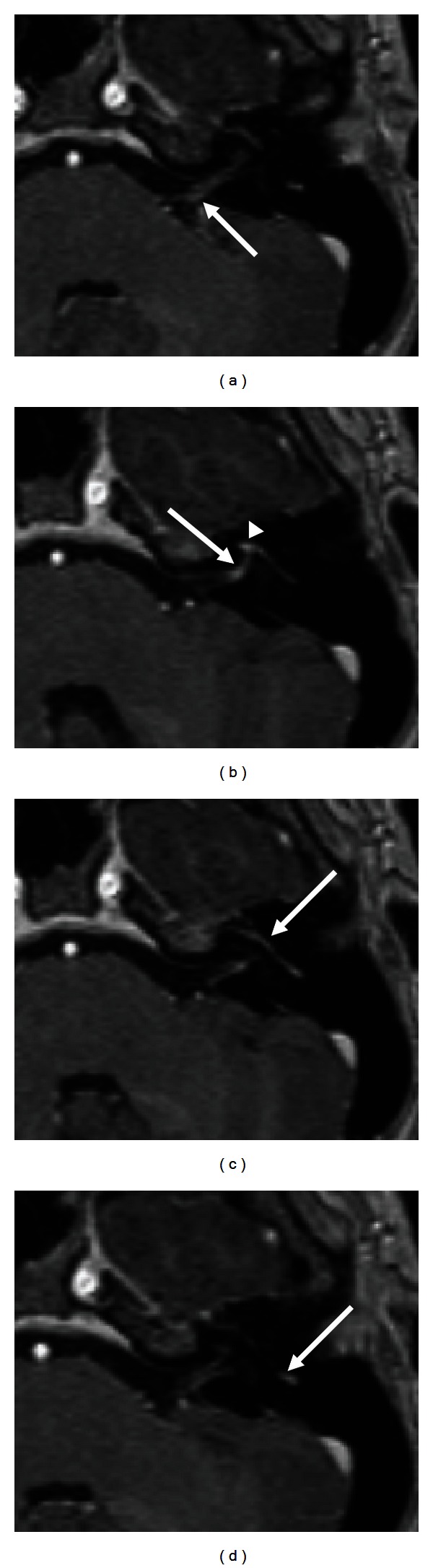
Bell's palsy. T1- weighted contrast-enhanced MR images demonstrate abnormal enhancement of the distal left cisternal (arrow, (a)), labyrinthine (arrow, (b)), first genu (arrowhead, (b)), and mastoid (arrow, (d)) segment of the facial nerve. Enhancement of the first genu, tympanic and mastoid segments was asymmetrically greater than the normal contralateral side (not shown).

 When gadolinium contrast is used, the normal facial nerve faintly enhances in the geniculate ganglion, tympanic, and mastoid segments (Figure 10) [7]. The cisternal, intracanalicular, labyrinthine, and parotid segments of the facial nerve do not normally enhance. Enhancement of the facial nerve in these regions raises suspicion of inflammatory or neoplastic processes. Asymmetric enhancement and/or thickening of the tympanic mastoid segments relative to the contralateral side should also be considered abnormal. For instance, in Bell's palsy, MRI with gadolinium contrast often demonstrates enhancement of the intracanalicular and labyrinthine segments of the facial nerve, as well as a greater degree of enhancement of the geniculate ganglion, tympanic, and mastoid segments (Figure 4) [7].

MRI can also reveal enlargement of the facial nerve, as may be seen in a neoplastic process. Particularly in the areas outside of the bony fallopian canal, this enlargement can be missed in high-resolution temporal bone CT. Facial nerve schwannomas may appear as fusiform masses in the labyrinthine and mastoid segments. In the cisternal, intracanalicular, and tympanic segments, schwannomas can appear lobulated (Figure 11). These lesions show strong enhancement with gadolinium on T1-weighted images. On CISS images, facial schwannomas will appear as nodular lesions along the nerve within the CPA or IAC. When large enough, they can fill the diameter of the internal auditory canal and may be difficult to differentiate from schwannomas of the vestibulocochlear nerve (Figure 11) [8].

 MRI characteristics can be used to distinguish masses around the facial nerve that require surgical excision from those that should not be surgically removed until facial function has been affected. For instance, lipomas of the internal auditory canal and first genu will appear hyperintense on T1-weighted imaging without additional gadolinium and can be reliably identified with T1-weighted imaging with fat saturation (Figure 12). Hemangiomas of the facial nerve typically occur at the geniculate ganglion and show avid contrast enhancement. Surgical removal of lipomas or hemangiomas often results in facial paralysis and so is typically only performed when preoperative facial function is poor [15, 16]. In contrast, cholesteatomas in the petrous apex classically appear hypointense on T1, hyperintense on T2, and show no or little contrast enhancement. Further confirmation of presence of cholesteatoma can be made with diffusion-weighted imaging, which reveals significantly decreased diffusion within the lesion (Figure 13) [17]. Cholesteatomas should be surgically removed when identified to avoid future decrease in facial function as they increase in size [18]. 

Reconstructed sagittal oblique CISS images through the internal auditory canals obtained perpendicular to the course of the IAC enable the evaluation of the caliber of the facial nerve. In patients with Moebius syndrome, CISS imaging is particularly useful in confirming the absence or small caliber of the facial nerve within the CPA and IAC (Figure 14).

For patients with hemifacial spasm, a loop of the anterior inferior cerebellar artery, posterior inferior cerebellar artery, or vertebral artery compresses the ipsilateral facial nerve at the root exit zone, leading to involuntary contractions of the facial musculature. High-resolution T2-weighted or CISS images can directly visualize the vascular loop and compressed facial nerve (Figure 15). 

### 3.3. Ultrasound

 In a recent study, ultrasound has been utilized to predict facial nerve outcomes in Bell's palsy. In this prospective, controlled study, patients with Bell's palsy, ultrasound was performed 2–7 days after the onset of paralysis using a General Electric Logiq 7 Pro with a 5 to 10 MHz linear array transducer [1]. Facial nerve diameter was measured proximally at the stylomastoid foramen, distally just proximal to the pes anserinus, and midway between these two points. The average diameter of the facial nerve was calculated using these three measurements and then compared with blink reflex studies and nerve conduction studies. A normal ultrasound measurement on the affected side had a 100% positive predictive value for normal facial function recovery at 3 months, a value significantly higher than values for nerve conduction (72–80%) and blink reflex studies (90%). Abnormal facial nerve ultrasound had a negative predictive value of 77% of House-Brackmann Grade II or worse facial nerve function at 3 months. This was again higher than that of nerve conduction studies (25–35%) and blink reflex studies (33%). 

### 3.4. Diffusion Tensor Tractography

In cases of large vestibular schwannomas, it can be difficult to distinguish between the facial nerve and the tumor on MRI. Both the facial nerve and the schwannoma have similar signal intensities, and larger tumors cause thinning of the facial nerve, making the nerve even more difficult to identify. Additionally, there is typically no intervening cerebrospinal fluid between the schwannoma and the facial nerve [2]. In these cases, diffusion tensor (DT) tractography may be useful for assessing facial nerve course and displacement. Taoka et al. [2] evaluated the accuracy of facial nerve DT tractography in 8 patients using a 1.5 Tesla scanner and a single-shot echo-planar sequence. Tracts of the facial nerve were determined from the pons to the internal auditory meatus using the DT images and compared with the facial nerve course visualized on high-resolution, heavily T2-weighted sequence of the brainstem. Tractography images were also compared with the facial nerve course observed intraoperatively. In 7 of 8 patients with tumors greater than 20 mm in size and whose facial nerves were indistinguishable from tumor on conventional imaging, DT tractography successfully visualized the facial nerve from the pons to the internal auditory meatus [2].

 Chen et al. [19] used a 3 Tesla scanner to obtain 3-dimensional (3D) visualization of the facial nerve in patients with vestibular schwannoma. DT images were acquired with an echo-planar/spin-echo sequence, and T1 anatomic axial images were used to construct a 3D tumor model. Detailed anatomy of the fibers was better visualized in larger tumors, with small fibers seen coursing inferior to the tumor from the porus acusticus to the brainstem. For the smallest tumor, the VII/VIII complex was visualized at the porus, but the cisternal segment of the facial nerve could not be seen with as much detail. However, the authors' ability to distinguish between cranial nerves VII and VIII was limited due to their proximity and similar size. 

Overall, DT tractography shows potential in evaluation of the course of the facial nerve, particularly in cases involving large vestibular schwannomas. Currently, this technique is computationally intensive and not automated, typically requiring intensive involvement of Ph.D. level personnel in the construction of these images. Further technological advancements in sensitivity and automation of the technique will likely lead to its greater clinical use.

## 4. Disorders of the Facial Nerve

The facial nerve can be affected by a number of different disorders resulting in weakness or paralysis of the facial musculature (Table 1). One of the critical steps in the clinical evaluation of facial paralysis is discerning whether a central nervous system process (cerebrovascular accident, brain tumor, and multiple sclerosis) or peripheral disease (Bell's palsy, middle ear infection/cholesteatoma, and facial nerve tumor) is the cause of the weakness. Due to the bilateral innervation of the forehead musculature, forehead-sparing facial paralyses suggest a central pathology. Many of the common causes of peripheral facial paralysis (Bell's palsy, chronic ear disease, cholesteatoma, and schwannomas) affect the distal branches of the ipsilateral facial nerve equally. In contrast, malignancies of the parotid and other structures lateral to the mastoid tip may cause paralysis of only one or a few of the distal branches of the facial nerve. Paralysis caused by tumors may be preceded by facial twitching. Timing of paralysis with respect to rapidity of onset, patient age at onset, and associated symptoms can also narrow the differential diagnosis. These clinical insights guide further evaluation, including the choice of imaging modality. 

Facial dystonias and hyperkinetic states (hemifacial spasm, essential blepharospasm, and Meige's syndrome) can also occur. All of these pathologies are discussed in detail below.

### 4.1. Idiopathic Facial Paralysis

The most common cause of facial paralysis, Bell's palsy, is characterized by the sudden onset of facial weakness. It has been associated with the reactivation of herpes simplex type 1 in the geniculate ganglion, leading to inflammatory edema of the facial nerve [20]. Imaging studies are not typically indicated in the early evaluation of this disorder. However, within the first month of onset of paralysis, MRI of the brain and brainstem with gadolinium contrast demonstrates abnormal enhancement of the intracanalicular, labyrinthine, tympanic and mastoid segments and asymmetric enhancement of the tympanic, and mastoid segments of the nerve (Figure 4). The presence of a parotid mass, antecedent facial twitching, or other neurologic signs in addition to a unilateral facial paralysis is an indication for earlier imaging of unilateral facial paralysis. MRI imaging is indicated later in the course of Bell's palsy if full recovery of facial function does not occur within 9 months of onset [21]. Recently, ultrasound has been utilized to predict outcomes in Bell's palsy, as discussed above [1].

Several other idiopathic and inflammatory processes can cause facial paralysis. These include sarcoidosis, Guillain-Barre syndrome, and multiple sclerosis. These paralyses may have a more indolent onset than Bell's Palsy, which is characterized by full onset of weakness within 72 hours. Typically these processes will involve other cranial nerves or parts of the brain in addition to the facial nerve. These pathologies are best evaluated utilizing MRI with gadolinium enhancement [3, 22].

### 4.2. Infectious Disorders of the Facial Nerve

A variety of infectious disorders can affect the facial nerve. For instance, dehiscences of the fallopian canal can lead to facial paralyses in the setting of chronic or acute otitis media. These are best evaluated with high-resolution CT of the temporal bones. In cases where extension of the infection into the central nervous system is suspected, both high-resolution temporal bone CT and MRI of the brain with gadolinium will be indicated. Lyme disease is increasingly common in the United States and is the most common cause of acquired bilateral facial paralysis. MRI of the brain with gadolinium may be normal or may show bilateral enhancement of the facial nerves and, potentially, other nerves as well [23]. Ramsey-Hunt syndrome is the result of reactivation of the varicella zoster virus can lead to facial paralysis and vesicles in the ear, on the face, or on the palate. Accompanying symptoms include otalgia, hearing loss, and vertigo. Enhancement of the facial and trigeminal nerves and spinal trigeminal tract can be detected on MRI. However, MRI is not usually indicated in the evaluation of this disorder, as the diagnosis is typically clear from the clinical presentation.

### 4.3. Traumatic Injury to the Facial Nerve

 Traumatic injury to the facial nerve can occur at a variety of levels, from the brainstem to the distal periphery. Blunt force trauma from high-speed motor vehicle accidents can fracture the temporal bone, leading to either direct involvement of the fallopian canal and nerve injury or indirect injury via postconcussive injury and edema. Delayed facial palsy results from reactivation of herpes simplex type I virus within the geniculate ganglion and occurs 3–14 days after a traumatic injury [24–26]. In the setting of a critically ill, initially comatose patient, distinguishing delayed facial palsy from a potentially reversible injury to the nerve can be impossible based on clinical information alone. In this setting, imaging can provide crucial information that can impact patient management. 

**Figure 5:**
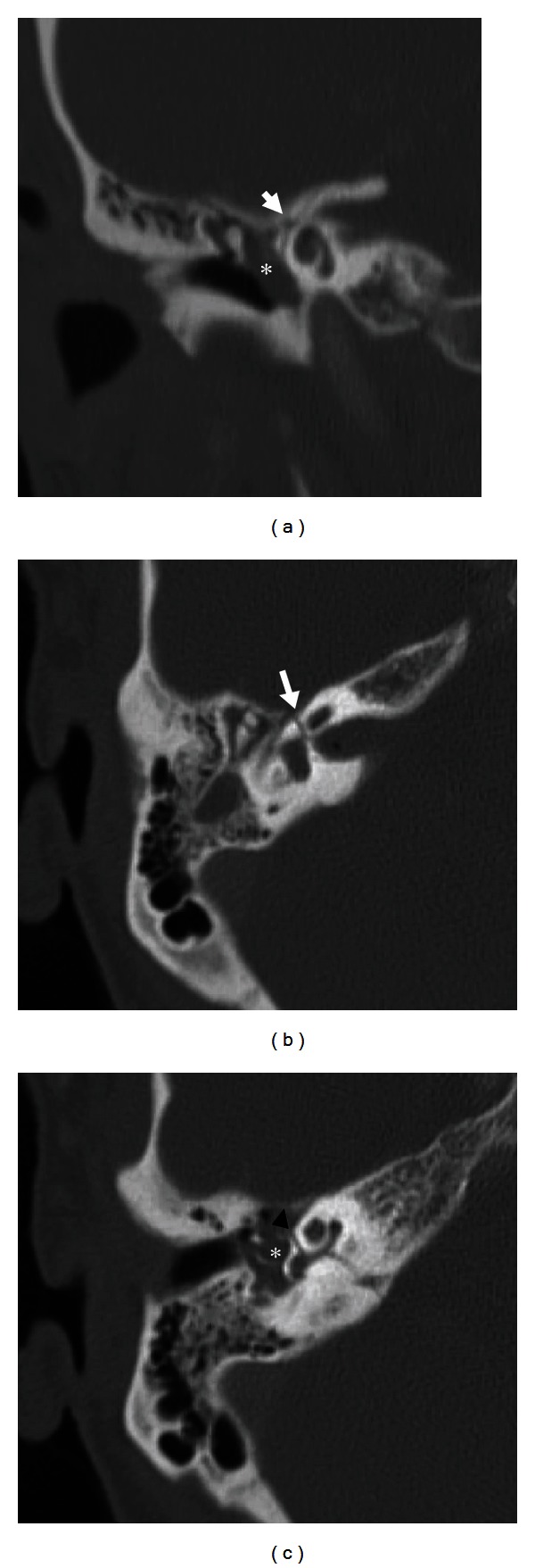
Transverse temporal bone fracture. Coronal (a) and axial (b) CT images demonstrate a transverse fracture through the labyrinthine segment of facial nerve canal (arrows). Axial CT image (c) shows the fracture involving the otic capsule and basal turn of the cochlea (arrowhead). Note blood products in the middle ear cavity (asterisks).

Facial nerve injuries more commonly occur in transverse fractures (38–50% of cases), rather than longitudinal fractures (20% of cases) of the temporal bone [3]. However, since longitudinal temporal bone fractures are much more common, they ultimately result in the highest number of facial injuries. Transverse fractures often affect the labyrinthine segment, while longitudinal fractures more often affect the geniculate ganglion, greater superficial petrosal nerve, tympanic segment, and mastoid segment (Figure 5). Potential mechanisms of direct facial injury following temporal bone fracture include fractures crossing through the facial canal, bony spicules impinging on the facial canal, or hematomas compressing the facial nerve. These are best visualized on noncontrast high-resolution CT of the temporal bones.

### 4.4. Congenital Facial Nerve Malformations

**Figure 6:**
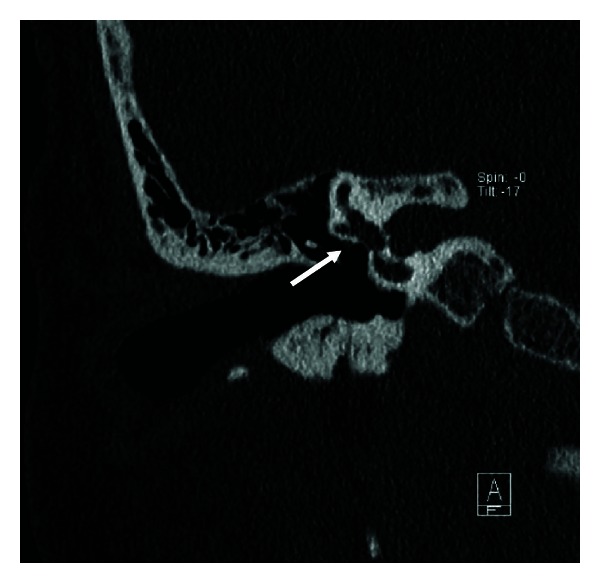
Facial nerve dehiscence. Coronal CT image of right temporal bone demonstrates dehiscence of right CN VII tympanic segment at the level of oval window (arrow).

**Figure 7:**
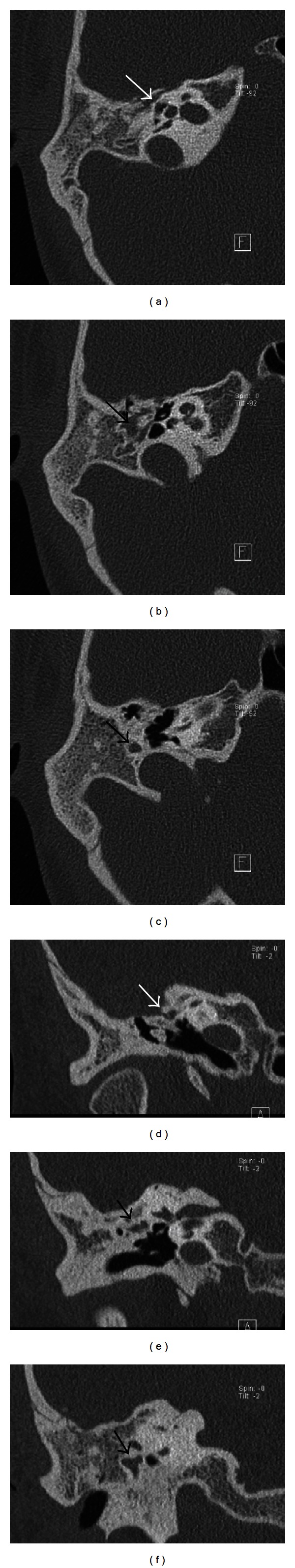
Aural atresia. Axial CT images (a)–(c) demonstrate abnormal facial nerve canal tympanic segment coursing posterolaterally to the middle ear cavity (arrows), instead of the normal posteromedial course. Coronal CT image (d) demonstrates the abnormal proximal tympanic segment of the facial nerve canal coursing superior to the middle ear cavity and ossicles (arrow), instead of its normal medial course. Coronal CT images (e)-(f) showing the abnormal distal tympanic segment and second genu of the facial nerve canal (arrows).

Congenital malformations of the facial nerve can clinically be asymptomatic or present with facial weakness. Bony dehiscences of the facial nerve canal are the most common of these and typically occur in the tympanic segment superior to the oval window (Figure 6). These dehiscences predispose the facial nerve to damage from inflammatory processes such as cholesteatoma and otitis media and are important to recognize prior to otologic surgery. Typically, these dehiscences are best visualized on high-resolution CT of the temporal bones [3]. In patients with aural atresia, the second genu and mastoid segment may be displaced anteriorly and laterally (Figure 7). The stylomastoid foramen may also be displaced anteroraterally in atretic ears, making the facial nerve more susceptible to intraoperative injury. High-resolution temporal bone CT can detect clinically significant changes in the course of the facial nerve prior to aural atresia repair.

 Congenital facial paralysis can occur as a result of facial nerve nucleus abnormalities in a variety of syndromes that include Moebius, DiGeorge, Goldenhar, CHARGE, trisomy 13, and trisomy 18. In Moebius syndrome, a constructive interference in steady-state (CISS) sequence can assist in early diagnosis by demonstrating the absence of the facial nerve [27]. Patients with CHARGE syndrome (colobomas, heart defects, choanal atresia, mental retardation, genitourinary anomalies, and ear anomalies) often have alterations in the course of the facial nerve. Posterior displacement of the labyrinthine segment is commonly seen [3]. Congenital hypoplasia or aplasia of the facial nerve can also occur as an isolated phenomenon and is best visualized by MRI. 

### 4.5. Vascular and Other Central Processes Affecting the Facial Nerve

A variety of central pathologies can affect the intracranial portion of the facial nerve, including cerebrovascular accident (CVA), brain tumors (primary and metastatic), and multiple sclerosis. Presentation of the paresis or paralysis is determined by the site of the intracranial lesion. Central paralyses occur when the lesion disrupts the facial nerve fibers prior to their decussation in the reticular formation of the caudal pons. Lesions affecting the facial nerve distal to the decussation can be mistaken for peripheral facial palsies; however, these are rare. Millard-Gubler syndrome is a mixed syndrome that is caused by lesions of the pons which lead to ipsilateral facial paresis, ipsilateral abducens paralysis, and contralateral hemiplegia. Millard-Gubler syndrome results from pathology disrupting the corticospinal tract prior to decussation plus the sixth and seventh nerve nuclei [28]. Eight-and-a-half syndrome results from involvement of the dorsal tegmen of the caudal pons involving the parapontine reticular formation or abducens nucleus and medial longitudinal fasciculus as well as the facial nerve nucleus. Symptoms of eight-and-a-half syndrome include intranuclear opthalmoplegia plus horizontal gaze palsy and ipsilateral facial paresis [29]. Regardless of the etiology of the lesion, these pathologies are best visualized on MRI.

Pontine infarcts comprise approximately 7% of all ischemic strokes. These infarcts are most frequently lacunar infarcts involving paramedian, short, and long circumferential perforators arising from the basilar artery [30, 31]. There are three main subtypes of pontine infarction: ventral, tegmental, and bilateral [31]. Ventral pontine infarcts are the most common type and are due to ischemia of the anteromedial and anterolateral pontine arteries, which supply the corticospinal tract, medial lemniscus, and cranial nerve nuclei. Clinically, patients present with severe ipsilateral paresis of the face, upper and lower extremities, dysarthria, and ipsilateral upper and lower extremity ataxia. Tegmental pontine infarcts result from ischemia of the short circumferential arteries which supply the tegmental area of the middle-upper pons. Patients present with eye movement disturbances, lemniscal and spinothalamic sensory deficits, and ipsilateral palsies of cranial nerves V, VI, and VII. Bilateral pontine infarcts are the least common and are associated with multiple brainstem infarcts. These patients present with pseudobulbar palsy and bilateral motor deficits of upper and lower extremities in addition to the signs seen in tegmental pontine infarction [31].

Central facial paralysis can also follow a CVA in the territory of the middle cerebral artery (MCA) or anterior cerebral artery (ACA) [32]. In the case of MCA stroke, patients can present with contralateral upper and lower extremity hemiparesis, contralateral hemianesthesia, and eye deviation towards the side of the infarct. When there is ischemia of the insula and operculum, forehead-sparing facial weakness also occurs [33]. For ACA strokes involving the dominant hemisphere, patients can present with contralateral-sided hemiparesis, contralateral hemianesthesia, dysarthria, aphasia, apraxia, and contralateral forehead-sparing facial paralysis. 

MRI with multiple sequences can accurately detect ischemic changes in patients with acute neurologic deficits. The following sequences are typically included: diffusion weighted imaging (DWI), T2-weighted (T2W) and fluid-attenuated inversion recovery (FLAIR), MR angiography (MRA), perfusion-weighted imaging (PWI), and gradient-recalled echo (GRE). DWI sequence can detect acute ischemia within minutes of onset as a focal area of hyperintensity [34], while T2W and FLAIR can detect acute ischemia within the first 3–8 hours after onset. Noncontrast time-of-flight MRA is used to evaluate the intracranial arterial system. PWI sequence with gadolinium contrast detects areas of the brain that have decreased cerebral blood flow but have not yet been severely injured. GRE sequences are tailored to be sensitive to the detection of iron products, thereby indicating the presence of prior hemorrhage [34]. 

### 4.6. Facial Dystonias

Patients with hemifacial spasm suffer unilateral twitching or spasms of facial muscles. Tortuosity of the anterior inferior cerebellar artery, posterior inferior cerebellar artery, basilar artery, or vertebral artery can compress the facial nerve at the root exit zone, resulting in unilateral spasms. MRI can identify the artery compressing the facial nerve and, thus, serve as a guide for microvascular decompression. In essential blepharospasm, involuntary blinking occurs with increased frequency, particularly in response to stimuli such as wind, sunlight, noise, or stress [35]. The etiology is unknown, although it is hypothesized to result from disinhibition of blinking due to a disorder of the basal ganglia [36]. In clinically equivocal cases, MRI can assist in diagnosis by excluding vascular compression of the facial nerve in the posterior cranial fossa. Lastly, Meige's syndrome is a rare disorder characterized by blepharospasm and involuntary contractions of the muscles in the jaw and tongue (oromandibular dystonia). MRI is used to exclude intracranial pathology in the evaluation of this idiopathic disorder. 

### 4.7. Neoplastic Processes Affecting the Facial Nerve

**Figure 8:**
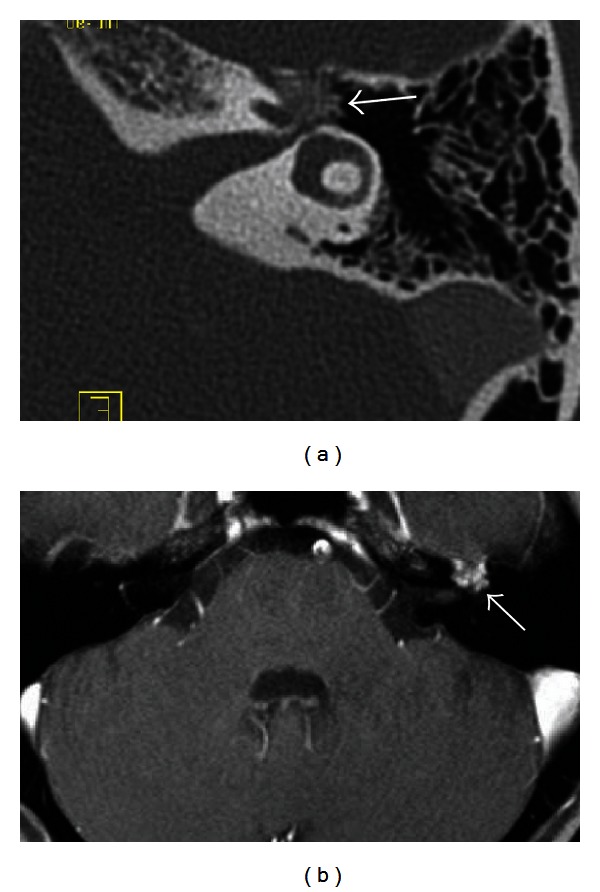
Facial nerve hemangioma. Axial CT (a) demonstrates a mass with trabecular bony matrix centered in the first genu of the facial nerve (arrow). Axial T1 postcontrast fat-saturated MRI (b) demonstrates the enhancing hemangioma (arrow).

**Figure 9:**
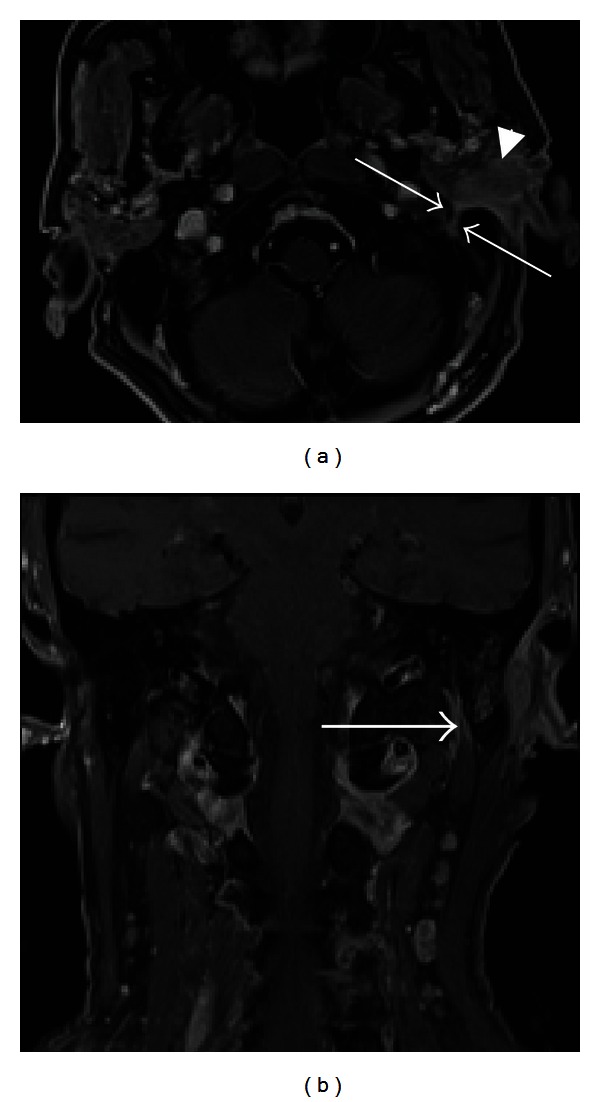
Perineural tumor spread along CN VII. Axial T1 (a) and coronal (b) postcontrast fat-saturated images demonstrate an enlarged, enhancing left facial nerve in the stylomastoid foramen and mastoid segment of the facial nerve canal (arrows), indicating perineural tumor spread from invasive squamous cell carcinoma (arrowhead) of the left external auditory canal.

**Figure 10:**
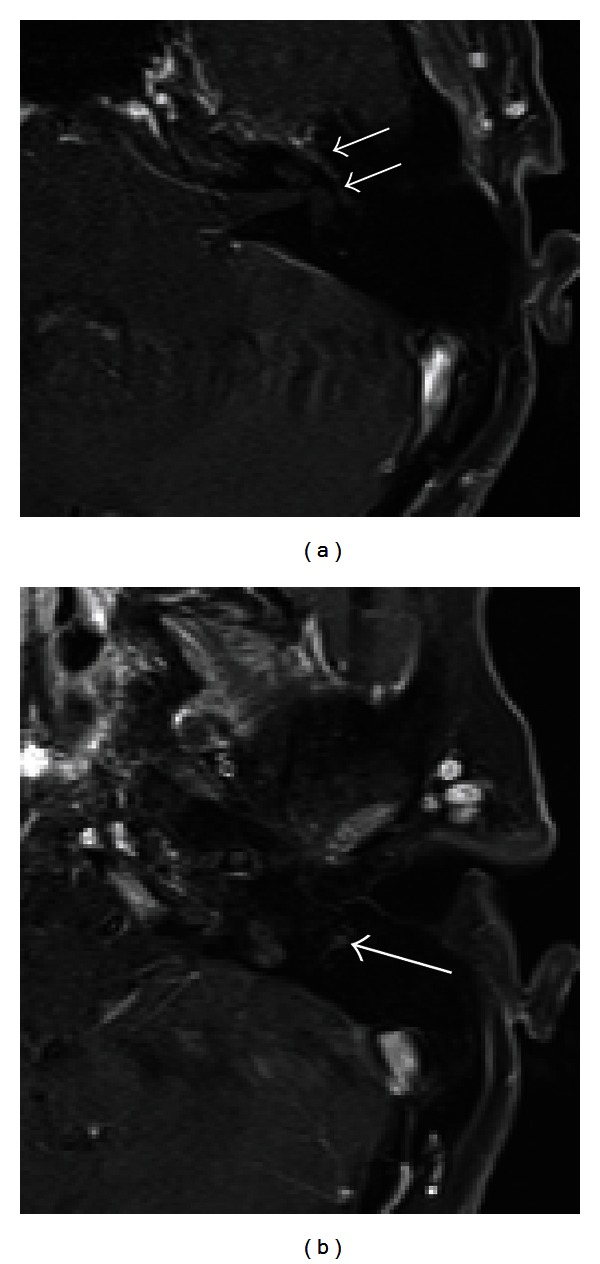
Normal facial nerve on MRI. Postcontrast fat-saturated T1-weighted MRI images of normal enhancing tympanic (a) and mastoid (b) CN VII segments.

**Figure 11:**
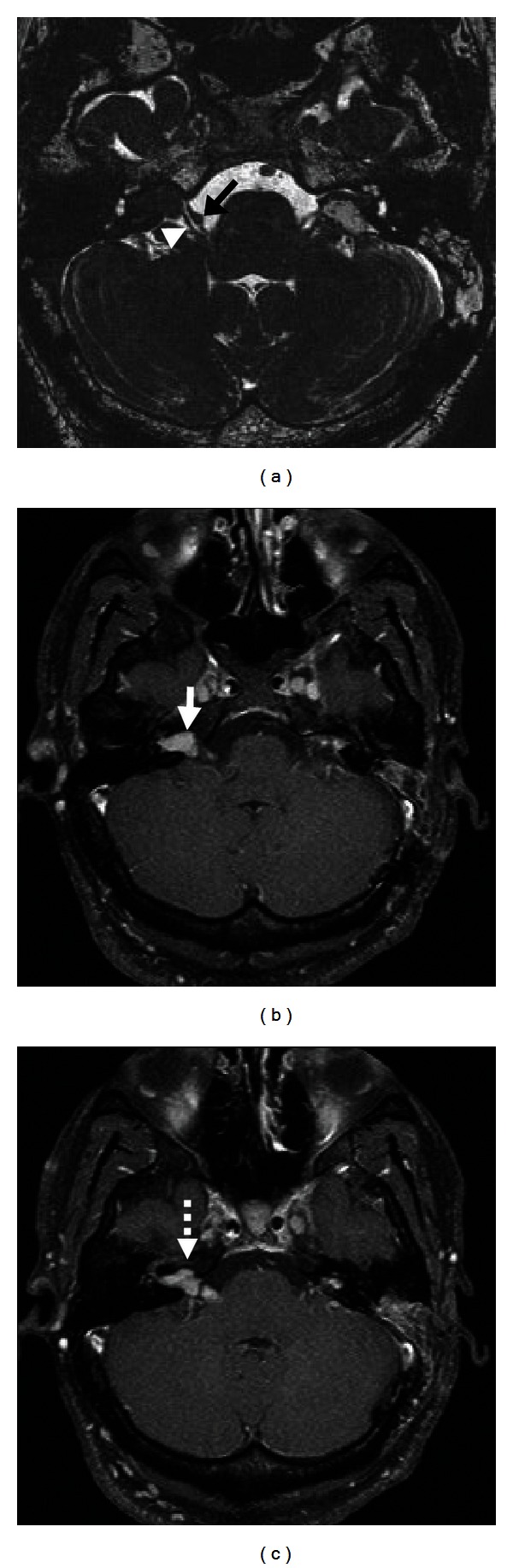
Right CN VII schwannoma in the cerebellopontine angle (CPA) in a patient with neurofibromatosis type II. Axial CISS sequence (a) demonstrates a small lesion along CNVII (black arrow), which courses anterior to CNVIII (white arrowhead) in the right CPA cistern. Axial T1 postcontrast fat-saturated images (b)-(c) demonstrate the enhancement of the right facial schwannoma (solid white arrow, (b)). Also note the vestibular schwannoma in the right IAC (dashed white arrow, (c)) and residual enhancement with postsurgical changes in the region of the left IAC.

**Figure 12:**
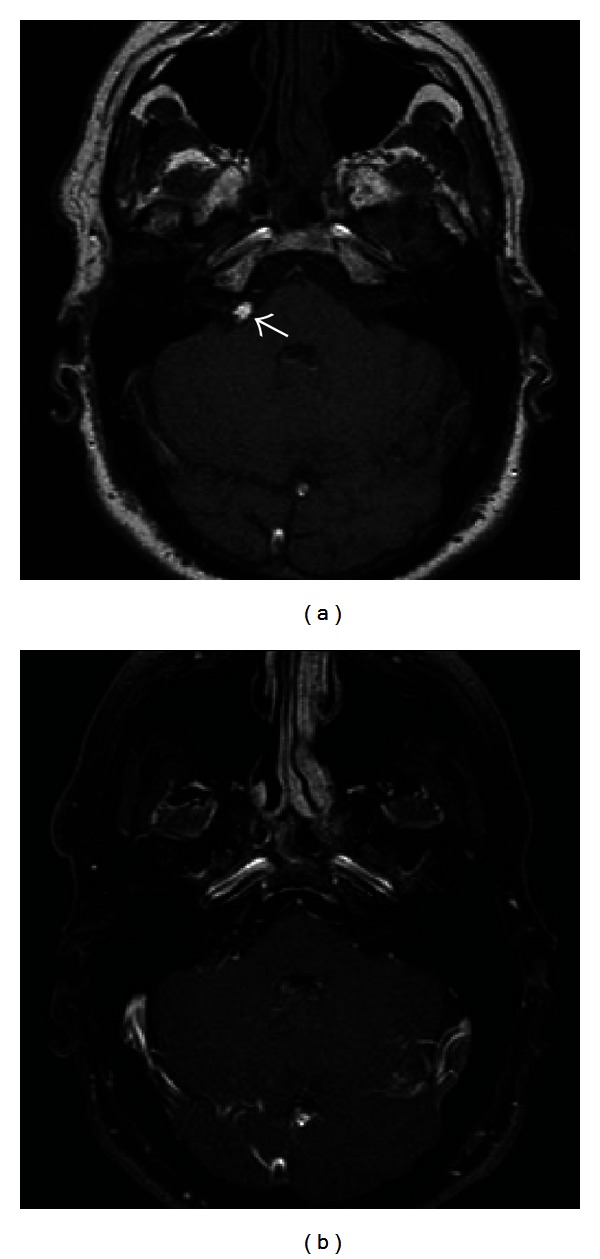
CPA Lipoma. Noncontrast T1-weighted image (a) demonstrates a globular hyperintense lesion in the right CPA (white arrow). (b) Postcontrast fat-saturated T1-weighted MR image shows signal dropout indicating that this lesion is composed of fat. There is no associated enhancement of this lesion.

**Figure 13:**
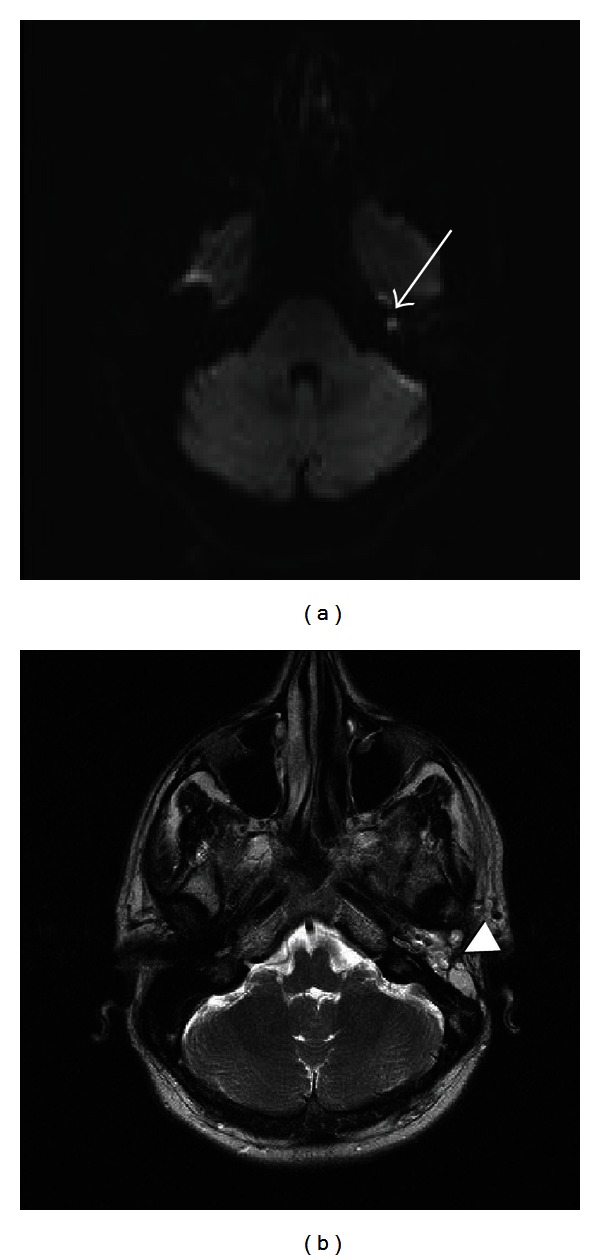
Recurrent cholesteatoma. Axial diffusion-weighted image (a) demonstrates abnormal hyperintense signal in the left temporal bone consistent with a focus on recurrent cholesteatoma (white arrow). Axial T2-weighted sequence (b) shows fat packing material in the left temporal bone (arrowhead) following mastoidectomy with blind sac closure.

**Figure 14:**
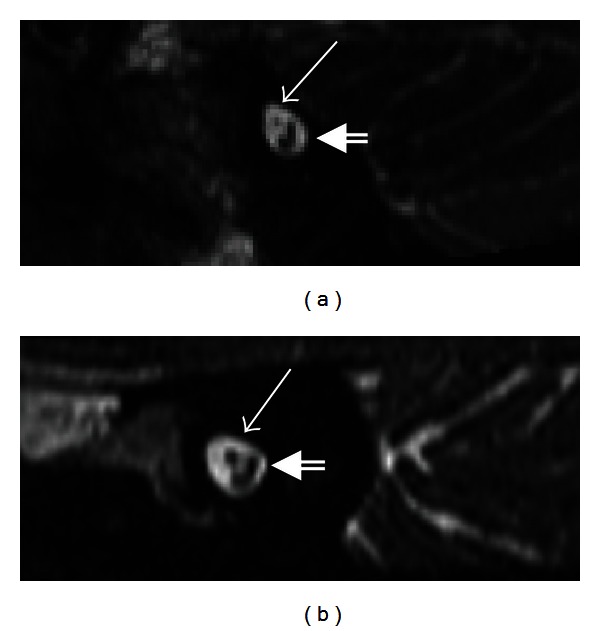
Moebius syndrome. Sagittal oblique CISS sequence of the internal auditory canal in a patient with Moebius syndrome (a) demonstrates the small caliber of the hypoplastic facial nerve in the anterior superior aspect of the canal (arrow). The normal vestibulocochlear nerve is seen posteriorly (double lined arrow). Sagittal oblique CISS image in a normal patient (b) demonstrates the normal caliber of the facial (solid arrow) and vestibulocochlear (double lined arrow) nerves for comparison.

**Figure 15:**
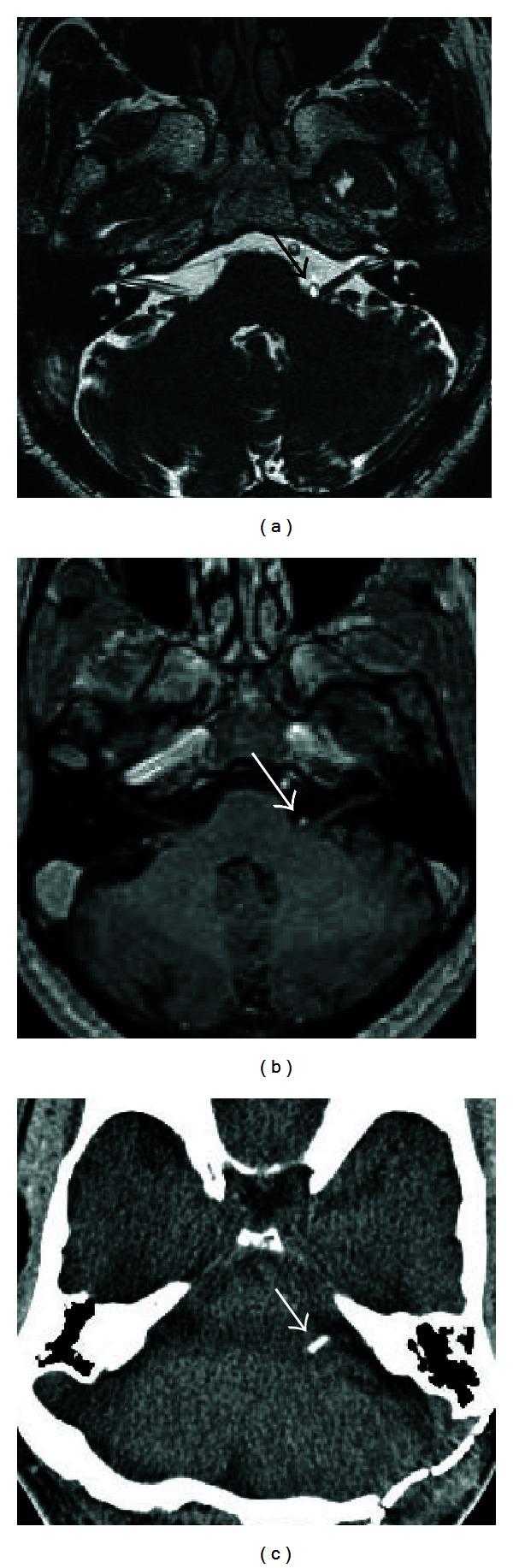
Neurovascular compression in a patient presenting with left hemifacial spasm. Axial CISS (a) and T1 postcontrast MRI (b) images demonstrate the left vertebral artery (arrow) abutting the left facial nerve at the root entry zone. Axial noncontrast CT scan (c) demonstrates the hyperdense postsurgical material from microvascular decompression surgery.

A variety of neoplastic processes can affect facial nerve function. Schwannomas can occur anywhere along the course of the facial nerve. Most commonly, they arise in the perigeniculate, tympanic, or mastoid segments. Lipomas can occur in the CPA or IAC, entrapping unmyelinated facial nerve fibers. Hemangiomas arise from the vascular plexus surrounding the facial nerve, most commonly in the perigeniculate region. Less commonly, these lesions are found in the IAC or mastoid segment [37]. Hemangiomas cause facial nerve dysfunction by direct neural compression or neural ischemia due to vascular steal [37]. Hemangiomas located in the geniculate ganglion can cause facial nerve weakness; tumors in the IAC may lead to progressive sensorineural hearing loss with poor speech discrimination [38]. Temporal bone CT can visualize mineralization of ossifying hemangiomas, distinguishing these tumors from facial nerve schwannomas (Figure 8). MRI is useful for proper identification of schwannomas, lipomas, and hemangiomas.

Paragangliomas are highly vascular tumors that originate in the paraganglionic tissue of the carotid bifurcation (carotid body tumors), jugular foramen (glomus jugulare), vagus nerve (glomus vagale), and tympanic plexus on the promontory (glomus tympanicum). They can occur sporadically or in association with tumor syndromes such as multiple endocrine neoplasia type II (MEN II), von Hippel-Lindau syndrome, or neurofibromatosis type I. Rare cases of facial nerve paragangliomas occurring along the mastoid segment have also been reported [39]. CT and MRI are essential for defining the extent of these lesions and for surgical planning. On contrast-enhanced T1-weighted MRI, the lesions exhibit a “salt-and-pepper” appearance; they are hyperintense on T2. Unlike cholesteatomas, they enhance with contrast [39]. 

Neoplasms in the middle ear can infiltrate or compress the tympanic segment of the facial nerve. These lesions include adenomas, schwannomas, and paragangliomas. Adenomas arise from the glands of the middle ear mucosa and often present as a middle ear mass with conductive hearing loss. CT typically shows a middle ear mass without evidence of bony erosion. 

 Malignant tumors of the parotid can affect the extratemporal facial nerve. Primary malignant neoplasms include mucoepidermoid carcinoma, adenoid cystic carcinoma, adenocarcinoma, malignant mixed tumors, acinic cell carcinoma, lymphoma, and squamous cell carcinoma. In particular, adenoid cystic carcinoma frequently exhibits perineural invasion (70–75%) [40]. Head and neck primaries may metastasize to intraparotid lymph nodes, which provide lymphatic drainage to the scalp, face, external auditory canal, and tympanic membrane. Skin tumors such as squamous cell carcinoma and melanoma account for 80–90% of parotid metastases [41]. MRI with gadolinium is useful for detecting perineural spread from parotid and minor salivary gland malignancies along the facial nerve.

## 5. Conclusion

Imaging can provide critical information for diagnosis and treatment of facial nerve disorders. MRI of the brain and brainstem is most useful for central pathologies affecting the facial nerve as well as lesions of the facial nerve proximal to the porus acusticus. High-resolution CT of the temporal bone best visualizes the course of the nerve within the fallopian canal to the stylomastoid foramen. Soft tissue CT with contrast or MRI can be used to evaluate areas of the distal course of the facial nerve within the parotid and soft tissues of the face. Facial nerve ultrasound may be useful in predicting functional outcomes 3 months after the onset of paralysis in patients with Bell's palsy. DT tractography is a new modality that shows promise for 3D visualization of facial nerve fibers, potentially lowering the risk of facial nerve injury during treatment of vestibular schwannomas.

## Figures and Tables

**Table 1:** Disorders of the facial nerve.

Infectious	Acute OM
	Chronic OM
	Cholesteatoma
	Herpes zoster oticus
	Lyme disease

Traumatic	Temporal bone fracture
	Iatrogenic injury
	Avulsion at brainstem
	Penetrating trauma

Neoplastic	Facial schwannoma
	Vestibular schwannoma
	Hemangioma
	Lipoma
	Glomus tumors
	Malignancy of skin/parotid

Congenital	Moebius syndrome
	Displacement (atresia)
	Aural atresia
	CHARGE syndrome
	Dehiscence of FC

Vascular	MCA infarct
	Pontine artery infarct
	Lacunar infarct

Idiopathic	Bell's palsy
	Multiple sclerosis
	Sarcoidosis

Inflammatory	Guillain-Barre

OM: otitis media; FC: fallopian canal; and MCA: middle cerebral artery.

## References

[1] Lo YL, Fook-Chong S, Leoh TH (2010). High-resolution ultrasound in the evaluation and prognosis of Bell’s palsy. *European Journal of Neurology*.

[2] Taoka T, Hirabayashi H, Nakagawa H (2006). Displacement of the facial nerve course by vestibular schwannoma: preoperative visualization using diffusion tensor tractography. *Journal of Magnetic Resonance Imaging*.

[3] Raghavan P, Mukherjee S, Phillips CD (2009). Imaging of the facial nerve. *Neuroimaging Clinics of North America*.

[4] Cisneros A, Orozco JRW, Nogues JAO (2011). Development of the stapedius muscle canal and its possible clinical consequences. *International Journal of Pediatric Otorhinolaryngology*.

[5] May M, Schaitkin BM (2000). *The Facial Nerve*.

[6] Curtin HD, Sanelli PC, Som PM, Som PM, Curtin HD (2003). Chapter 19 temporal bone: embryology and anatomy. *Head and Neck Imaging*.

[7] Al-Noury K, Lotfy A (2011). Normal and pathological findings for the facial nerve on magnetic resonance imaging. *Clinical Radiology*.

[8] Jäger L, Reiser M (2001). CT and MR imaging of the normal and pathologic conditions of the facial nerve. *European Journal of Radiology*.

[9] Veillon F, Ramos-Taboada L, Abu-Eid M, Charpiot A, Riehm S (2010). Imaging of the facial nerve. *European Journal of Radiology*.

[10] Liu L, Arnold R, Robinson M (2012). Dissection and exposure of the whole course of deep nerves in human head specimens after decalcification. *International Journal of Otolaryngology*.

[11] Crawford SC, Harnsberger HR, Swartz JD, Swartz JD, Harnsberger HR (1998). Chapter 7: the facial nerve (cranial nerve VII). *Imaging of the Temporal Bone*.

[12] Nager GT, Proctor B (1991). Anatomic variations and anomalies involving the facial canal. *Otolaryngologic Clinics of North America*.

[13] Yellon RF, Branstetter BF (2010). Prospective blinded study of computed tomography in congenital aural atresia. *International Journal of Pediatric Otorhinolaryngology*.

[14] Mu X, Quan Y, Shao J, Li J, Wang H, Gong R (2012). Enlarged geniculate ganglion fossa: CT sign of facial nerve canal fracture. *Academic Radiology*.

[15] Brodsky JR, Smith TW, Litofsky S, Lee DJ (2006). Lipoma of the cerebellopontine angle. *American Journal of Otolaryngology*.

[16] Isaacson B, Telian SA, McKeever PE, Arts HA (2005). Hemangiomas of the geniculate ganglion. *Otology and Neurotology*.

[17] De Foer B, Vercruysse JP, Spaepen M (2010). Diffusion-weighted magnetic resonance imaging of the temporal bone. *Neuroradiology*.

[18] Yorgancilar E, Yildirim M, Gun R (2013). Complications of chronic suppurative otitis media: a retrospective review. *European Archives of Oto-Rhino-Laryngology*.

[19] Chen DQ, Quan J, Guha A, Tymianski M, Mikulis D, Hodaie M (2011). Three-dimensional in vivo modeling of vestibular schwannomas and surrounding cranial nerves with diffusion imaging tractography. *Neurosurgery*.

[20] Kennedy PG (2010). Herpes simplex virus type 1 and Bell’s palsya current assessment of the controversy. *Journal of NeuroVirology*.

[21] Lanser MJ, Jackler RK (1991). Gadolinium magnetic resonance imaging in Bell’s palsy. *Western Journal of Medicine*.

[22] Pickuth D, Heywang-Kobrunner SH (2000). Neurosarcoidosis: evaluation with MRI. *Journal of Neuroradiology*.

[23] Vanzieleghem B, Lemmerling M, Carton D (1998). Lyme disease in a child presenting with bilateral facial nerve palsy: MRI findings and review of the literature. *Neuroradiology*.

[24] Salvinelli F, Casale M, Vitaliana L, Greco F, Dianzani C, D’Ascanio L (2004). Delayed peripheral facial palsy in the stapes surgery: can it be prevented?. *American Journal of Otolaryngology*.

[25] Gianoli GJ (2002). Viral titers and delayed facial palsy after acoustic neuroma surgery. *Otolaryngology*.

[26] Brackmann DE, Fisher LM, Hansen M, Halim A, Slattery WH (2008). The effect of famciclovir on delayed facial paralysis after acoustic tumor resection. *Laryngoscope*.

[27] Verzijl HTFM, Valk J, De Vries R, Padberg GW (2005). Radiologic evidence for absence of the facial nerve in Möbius syndrome. *Neurology*.

[28] Silverman IE, Liu GT, Volpe NJ, Galetta SL (1995). The crossed paralyses. The original brain-stem syndromes of Millard-Gubler, Foville, Weber, and Raymond-Cestan. *Archives of Neurology*.

[29] Eggenberger E (1998). Eight-and-a-half syndrome: one-and-a-half syndrome plus cranial nerve VII palsy. *Journal of Neuro-Ophthalmology*.

[30] Saia V, Pantoni L (2009). Progressive stroke in pontine infarction. *Acta Neurologica Scandinavica*.

[31] Bassetti C, Bogousslavsky J, Barth A, Regli F (1996). Isolated infarcts of the pons. *Neurology*.

[32] Cattaneo L, Saccani E, De Giampaulis P, Crisi G, Pavesi G (2010). Central facial palsy revisited: a clinical-radiological study. *Annals of Neurology*.

[33] Kim JYS, Narayan D (2012). Facial nerve parlaysis. *Medscape Reference*.

[34] Nentwich LM, Veloz W (2012). Neuroimaging in acute stroke. *Emergency Medicine Clinics of North America*.

[35] Spencer BR, Digre KB (2010). Treatments for neuro-ophthalmologic conditions. *Neurologic Clinics*.

[36] Boghen D, Tozlovanu V, Iancu A, Forget R (2002). Botulinum toxin therapy for apraxia of lid opening. *Annals of the New York Academy of Sciences*.

[37] Ahmadi N, Newkirk K, Kim HJ (2013). Facial nerve hemangioma: a rare case involving the vertical segment. *Laryngoscope*.

[38] Friedman O, Neff BA, Willcox TO, Kenyon LC, Sataloff RT (2002). Temporal bone hemangiomas involving the facial nerve. *Otology and Neurotology*.

[39] Kunzel J, Zenk J, Koch M, Hornung J, Iro H (2012). Paraganglioma of the facial nerve, a rare differential diagnosis for facial nerve paralysis: case report and review of the literature. *European Archives of Oto-Rhino-Laryngology*.

[40] Garden AS, Weber RS, Morrison WH, Ang KK, Peters LJ (1995). The influence of positive margins and nerve invasion in adenoid cystic carcinoma of the head and neck treated with surgery and radiation. *International Journal of Radiation Oncology Biology Physics*.

[41] Malata CM, Camilleri IG, McLean NR (1997). Malignant tumours of the parotid gland: a 12-year review. *British Journal of Plastic Surgery*.

